# Spherical CNN for Medical Imaging Applications: Importance of Equivariance in image reconstruction and denoising

**Published:** 2023-10-26

**Authors:** Amirreza Hashemi, Yuemeng Feng, Hamid Sabet

**Affiliations:** Athinoula A. Martinos Center for Biomedical Imaging, Department of Radiology, Massachusetts General Hospital & Harvard Medical School, Boston, MA, USA

**Keywords:** Equivariant Network, CNN, Image Reconstruction, Denoising, Medical Imaging

## Abstract

This work highlights the significance of equivariant networks as efficient and high-performance approaches for tomography applications. Our study builds upon the limitations of conventional Convolutional Neural Networks (CNNs), which have shown promise in post-processing various medical imaging systems. However, the efficiency of conventional CNNs heavily relies on an undiminished and proper training set. To tackle this issue, in this study, we introduce an equivariant network, aiming to reduce CNN’s dependency on specific training sets. We evaluate the efficacy of equivariant spherical CNNs (SCNNs) for 2- and 3-dimensional medical imaging problems. Our results demonstrate superior quality and computational efficiency of SCNNs in denoising and reconstructing benchmark problems. Furthermore, we propose a novel approach to employ SCNNs as a complement to conventional image reconstruction tools, enhancing the outcomes while reducing reliance on the training set. Across all cases, we observe a significant decrease in computational costs while maintaining the same or higher quality of image processing using SCNNs compared to CNNs. Additionally, we explore the potential of this network for broader tomography applications, particularly those requiring omnidirectional representation.

## I. INTRODUCTION

ARTIFICIAL intelligence (AI) has emerged as a promising technology in the medical imaging applications by enhancing diagnosis, treatment, and patient outcomes. It has shown immense capability to improve the quality of medical images in several ways such as enhancing spatial resolution, noise reduction, and lowering acquisition time [1–3]. Among the AI tools, convolutional neural networks (CNN) are distinctly recognized as a powerful tool in a broad range of medical imaging processing. For instance, CNN can learn features from the 2-dimensional (2-D) images and use them to reconstruct high-quality 3-dimensional (3-D) images. It is also reliable tool for image denoising while preserving the details of the images. In recent years, the use of CNN for reconstruction has extended to radiation-imaging modalities, such as PET, CT and SPECT which results in improvement of image quality and computational efficiency [4–6]. Thanks to recent progresses in deep learning CNN, this approach has evolved to produce high-quality images that are comparable to or better than images produced by conventional reconstruction algorithms such as maximum likelihood expectation maximization (MLEM) [7, 8]. However, the enhancement of conventional CNNs has been hampered by many existing limitations that include: overfitting, limited interpretability, limited ability to handle non-Euclidean spaces (e.g. image on the sphere) and missing or insufficient data, and being computationally expensive. The quality and quantity of data used for training directly impact the model’s ability to learn and generalize to new data. A well-designed and diverse training set can help the model to identify and extract features from local details of images, improving its performance on new and unseen data. In this regard, an insufficient or biased training set can lead to overfitting or underfitting, which can result in poor performance and reduced accuracy. Therefore, selecting an appropriate and representative training set is crucial for achieving reliable and robust results when using CNN. On the other hand, acknowledging that the symmetry is a crucial aspect of many anatomical structures and objects, and it is evident in medical images as well [9, 10]. These symmetrical representations can be leveraged in the creation of training set representation of neural network model. In this regard, recent studies [11–13] on AI image reconstruction of PET and SPECT have suggested that the inclusion of the symmetrical rotation of training data would enhance the image quality and lower the computational cost. However, the improvements of the recent studies were limited to stacked representation of a single rotation of the training set. Additionally, it has been shown that the rotational invariance of the denoising filters leads to promising outcomes, yielding high-quality image outputs [14]. Here, our objective is to extend upon the recent progress and show the advantage of equivariance in machine learning-based medical imaging processing. In machine learning of symmetrical systems, behavioral properties exhibit distinct transformation characteristics when subjected to translation, reflection, and rotation with respect to small local sample representatives. Equivariant neural networks ensure that their features maintain a predetermined transformation characteristics when the input undergoes transformations. We investigate the use of equivariant spherical CNN (SCNN) for medical imaging applications and particularly for problems where the representative domain is symmetrical (or spherical) such as brain image. We will show variational invariance of training set plays a key role on the performance of CNNs in medical imaging applications and therefore approaches such as SCNN are well suited because the network model is adapted to rotational, translational and permutational invariance. To prove our proposition, we will show the efficiency of SCNNs for denoising and reconstruction for several problems in 2- and 3-dimension (2- and 3-D). Additionally, we utilize the SCNN as a handicap method for the conventional method of MLEM to improve the performance and quality of the overall outcome based on the limited dataset. Ultimately, we show that SCNN and equivariant network is a viable option that reduces reliance of training set for CNN-based medical imaging algorithms.

## II. Method

SCNN was first introduced in [15–18] as a particular form of CNNs with a group representation called *irreducible representations* to efficiently encode the symmetries of data. By leveraging the properties of irreducible representations and spherical harmonics, SCNN can perform convolution operations on spherical space in a way that is invariant to rotations and permutations, making it well-suited for processing symmetrical data. In this regard, equivariant SCNN utilizes spherical harmonics and convolutional operations to efficiently extract features from the spherical signals. Spherical harmonics capture the harmonic components of the input signal, while convolutional operations perform localized computations on the spherical surface. In addition, the sphere has a constant positive curvature, which means that the distance between any two points on the sphere is always less than the distance between the same two points on a flat surface. This property allows SCNNs to be more efficient at processing data than conventional CNNs, which are designed for flat, only Euclidean spaces.

To achieve equivariance, SCNN applies a group-equivariant convolutional layer that preserves rotational symmetries. This layer ensures that the network’s output is invariant to rotations in the input space, enabling it to learn and recognize features independent of their orientation. The key underlying equation is the equivariance condition which represents how the network’s outputs in response to relations of the input data. Let *R*_*g*_ denote the rotation operator corresponding to a group element *g* in the group of relations. For an input signal *x* and an equivariance feature map *f* can be expressed as:

$$f(R_{g}\cdot x)=R_{g}\cdot f(x)$$

where this equation implies that the feature map *f* behaves consistently under rotations, and *R*_*g*_ acting both on the input and the output.

To achieve equivariance convolutions, let *Y*_*l,m*_ represents the spherical harmonic basis function with degree *l* and order *m*. The convolutional operator can be expressed as:

$$y_{i,j}=\Sigma_{l,m}\;w_{l,m}\cdot Y_{l,m}\;(R_{g}\cdot x_{i,j})$$

where *y*_*i,j*_ denotes the output feature map at spatial location (*i, j*), *w*_*l,m*_ are the learned convolutional weights associated with the spherical harmonics basis functions, and *x*_*i,j*_ represents the input signal at location (*i, j*).

Through training, equivariant SCNN optimizes its parameters, including the convolutional weights, using gradient-based optimization algorithms like backpropagation. The objective is to minimize a loss function that reflects the task at hand, such as cross-entropy for classification or mean squared error for regression. The training process can be outlined as minimizing a loss function *L* given a set of training samples (*x*_*i*_, *y*_*j*_):

$$min_{\theta }\Sigma_{i}L(R_{g}\cdot x_{i,j})$$

where *θ* denotes the network’s parameters, *f(x*_*i*_) represents the output of the network given input *x*_*i*_, and *y*_*j*_ is the corresponding ground truth. The SCNN architecture consists of multiple equivariant convolutional layers followed by non-linear activation functions and pooling operations. The extracted features are then processed by fully connected layers to perform classification or regression tasks.

### A. Network Implementations: SCNN and CNN in 2-D and SCNN in 3-D

Our 2-D SCNN is built of three layers that include input, hidden, and output layers with 8 rotational actions on the group space. Each layer is connected to the inner batch normalization and rectified linear unit (ReLU) activation. Input and hidden layers have *trivial* and *regular representative* functions respectively with a kernel size of 3 and the output layer has an irreducible representation function with kernel size of one (for the details of SCNN descriptions and more information on the representative functions, see [19]). For the sake of comparison, a 2-D deep learning conventional CNN is chosen with 5 layers (3 hidden layers), 64 channels, and a ReLU activation that was introduced in [20]. Our 3-D SCNN is built of three layers that include input, hidden, and output layers with theoretically unlimited rotational actions on the group space. Each layer is connected to the generalized 3-D batch normalization function followed by norm nonlinearity activation function. Input and hidden layers have *trivial* and *spectral-regular* representative functions with a kernel size of 3. And the output layer has an irreducible representation function with one kernel. Trivial representative means that input and output fields are both scalar and spectral regular representative is when the input and output fields exude Fourier coefficients. More details on the mathematical meanings of representatives are presented in [19, 21].

We utilized the *pytorch* framework [22] and the *escnn* package where the details are provided in [19, 21]. SCNN simulations were done on Linux Ubuntu v20.04 with an Intel Xeon E5–2687W 3.1GHz CPU, 128GB RAM, and an NVIDIA TITAN RTX GPU card with 24GB of memory.

### B. Machine Learning Assisted Image Reconstruction

#### I. Monte Carlo simulation setup

The simulations were conducted using GATE with a designed phantom comprising six regions of hot rods. These rods have a length of 5 mm and diameters measuring 6.5 mm, 5.5 mm, 4.5 mm, 3.5 mm, 2.2 mm, and 1.6 mm. Additionally, there is a central cold region with a diameter of 3 cm. The ratio between the hot rods and the warm background is set to 10, with the activity of the hot rods at 53kBq/cc and background at 5300 Bq/cc. The phantom is positioned in air, and a 10-minute acquisition is performed. The simulated whole-body PET geometry consists of 5 rings and each ring has 44 detector blocks. Each detector block consists of 2.1 × 2.1 × 20 mm3 pixels with 2.2 mm pixel pitch, two depth of interaction (DOI) levels. The reconstructed volume has a voxel size of 0.8 × 0.8 × 0.8 mm3. Three different target coincidence time resolutions (CTRs) for the system are tested: 50, 100, and 200 ps FWHM.

#### II. Machine learning framework

In this study, we employed a machine learning assisted reconstruction algorithm namely SCNN-assisted for image reconstruction. The algorithm consisted of two main steps. First, an early list mode MLEM reconstruction was performed based on the simulation data. Then, a subsequent SCNN reconstruction was carried out using the 2-D central slice as a subset of the MLEM reconstruction. The PET system matrix in list mode MLEM was calculated on the fly, taking into consideration the probability of detecting one coincidence event emitted from a voxel by the line of response connecting the opposing detector pixels.

## III. RESULTS

### A. Comparison between SCNN and CNN

#### I. Denoising Example for 2-D Data

**Fig. 2.**
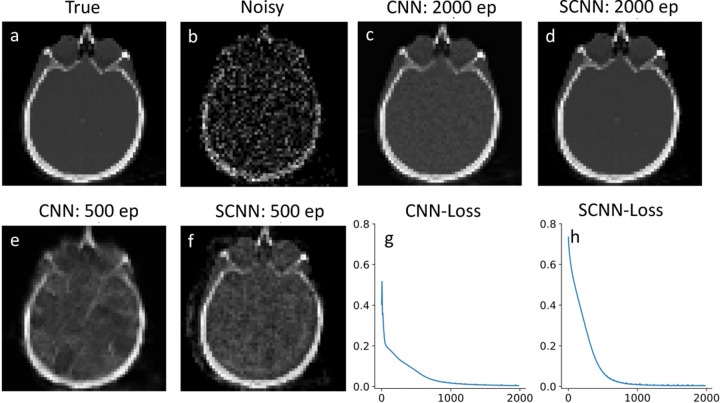
Denoising results of brain image. Plots a and b show true and noisy images, comparison between CNN and SCNN are shown in plots c-f for 2000 and 500 epochs. The loss functions for CNN and SCNN are shown in plots g and h respectively.

To evaluate the performance of our SCNN model, first we compare its results with those of conventional CNNs for denoising a noisy brain image. The training data for denoising is a brain image from a brain library of scikit-learn. Figure 2a presents the true image, while Figure 2b displays the corresponding noisy image with Poisson noise. Both CNN and SCNN models are trained for 2000 epochs, and the denoised images are shown in Figure 2c and Figure 2d at the 2000th epoch. Additionally, the denoised images at the 500th epoch are illustrated in Figure 2e and Figure 2f for CNN and SCNN, respectively. The loss function, measured by the mean square error (L2 norm), is employed to assess the training progress. The loss function versus epoch plots for CNN and SCNN are presented in Figure 2g and Figure 2h, respectively. Regarding the computational aspect, both CNN and SCNN models exhibit similar computational times for 2000 epoch iterations when executed on the GPU, measuring 104 seconds for CNN and 106 seconds for SCNN in the case of denoising.

#### II. Image reconstruction example

**Fig. 3.**
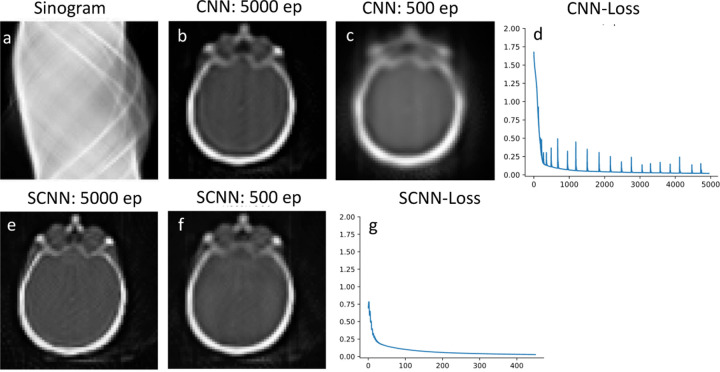
Reconstruction results using sinogram input that is shown in plot a. Comparison between the reconstructed image at 5000 and 500 epochs are shown in plots b and c for CNN and plots e and f for SCNN. Plots d and g are loss function vs epoch for CNN and SCNN respectively.

To evaluate the performance for image reconstruction, the sinogram of the benchmark brain image is generated using the Radon transform which is shown in Figure 3a, and the forward propagation model is integrated into the loss function of the neural networks. The reconstruction results for CNN and SCNN are presented for two epochs at 5000 and 500 iterations, while the corresponding loss function versus epoch plots are displayed in Figures 3d and 3g. The minimum values of the loss function for SCNN and CNN converge to 0.015 and 0.14, respectively. In terms of computational time, CNN takes 766 seconds, while SCNN requires 784 seconds to complete 5000 epochs. Additionally, the computational time for SCNN at 500 epochs is estimated to be 87 seconds.

#### III. Denoising Example for 3-D Data

**Fig. 4.**
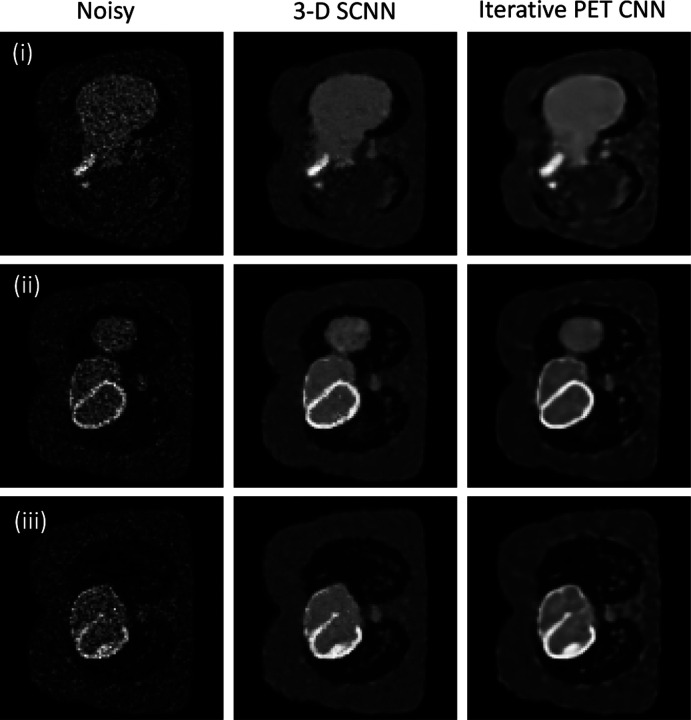
Comparison of 3-D SCNN for three selected slices (corresponding slices are placed in rows of i, ii, and iii) of the input noisy images, 3-D SCNN outputs and the outputs of iterative PET CNN reconstruction at 100^th^ iteration [24, 25].

For 3-D case, we examine our network with the simulation study of XCAT phantom [23] that is discussed in previous works on iterative PET image reconstruction using CNN representation [24, 25] where neural network model was embedded into the iterative image reconstruction framework. The dataset includes image matrix of the size 128×128×49 and the voxel size of 3.27×3.27×3.27 mm^3^, with the Poisson noise level that was generated to replicate the real-world dataset. The noisy results served as inputs for our denoising 3-D SCNN model. To facilitate comparison, we selected and presented three slices of the noisy inputs in Figure 4. Our 3-D SCNN model was exclusively initialized with the noisy dataset, and the denoising problem were run for 60,000 epochs until the loss value approached near zero. Additionally, we displayed the corresponding outputs of the image reconstruction obtained from iterative PET reconstruction, which was executed for 100 iterations and can be found in [26].

### B. SCNN-Assisted Efficient Image Reconstruction Based on Small Dataset

**Fig. 5.**
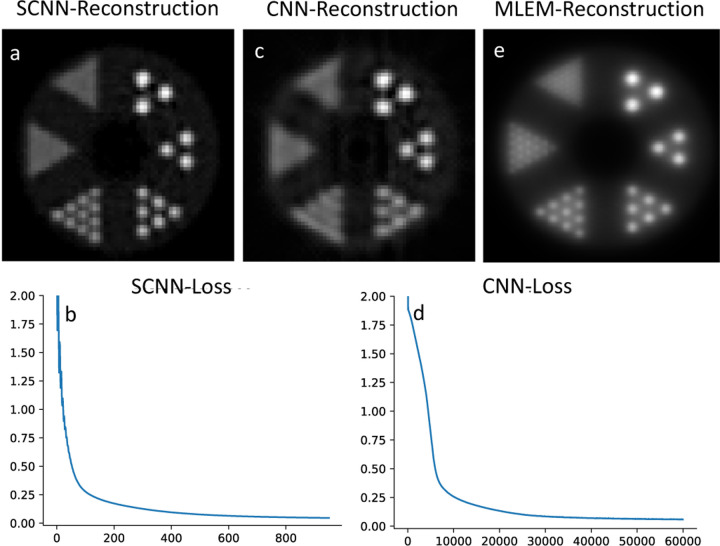
Results of SCNN assisted reconstruction is shown in plots a and, loss result of the first 1000 epochs of the loss result are shown in plot b. Results of CNN assisted reconstruction is shown in plots c and, loss result is shown in plot d. Plot e shows the MLEM reconstruction at second iteration.

Figure 5a and 5b display the outcomes of SCNN-assisted reconstruction, where the reconstruction process begins with the initial two iterations of MLEM-based reconstruction which is demonstrated in Figure 5e. Only a single central slice of the MLEM-reconstructed image and its sinogram is imported as a training set for the SCNN reconstruction algorithm. A similar framework is employed for CNN, and the results are presented in Figure 5c and 5d. The SCNN-assisted reconstruction demonstrates high accuracy in reconstructing rods with diameters ranging from 3.5 mm to 6.5 mm. However, the reconstruction appears blurry for the rods with diameters of 2.2 mm and 1.6 mm. It is worth noting that the SCNN loss function exhibits a consistent and steady decrease with minimal instability up to the point where the minimum loss value is achieved (Figure 5b).

## IV. Discussion

**Fig. 6.**
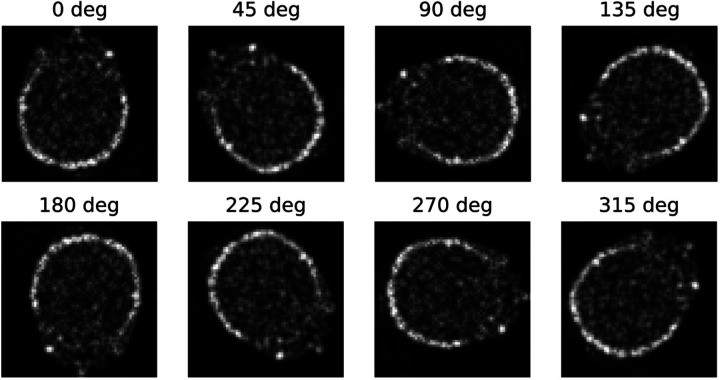
Rotational representative actions show output transforms of a noisy brain image.

The results obtained from 2-D SCNN demonstrate higher accuracy in both image denoising and reconstruction problems compared to conventional CNNs. This improvement can be attributed to the utilization of an equivariant dataset as input for SCNN, which is not present in conventional CNNs. To illustrate this point, Figure 6 showcases eight equivariant rotational representative actions applied to denoising cases throughout a single epoch iteration of SCNN. Consequently, each training input undergoes transformation within the rotational space of these representative actions. This inclusion of equivariant representative actions reduces SCNN’s dependence on the completeness of its training set, leading to enhance the output accuracy. For instance, in previous CNN-based works [5, 24, 25, 27] different rotations and translations of the input datasets were added to the training sets which make the process arbitrary to the developer’s experience and the designated dataset.

Also, SCNN exhibits faster convergence, reaching a near-zero value for the loss function in fewer iterations. Specifically, in the 2-D denoising case, SCNN achieves a loss value equivalent to that of CNN at 2000 epochs by only 1000 epochs. For the 2-D reconstruction case, SCNN’s loss value converges to a lower value of 0.02 at 500 epochs, whereas CNN requires 5000 epochs to converge to a higher value of 0.14. Figure 3b and 3f provide a visual comparison, demonstrating that SCNN achieves higher quality image reconstruction ten times faster in terms of epoch count and nearly nine times faster in terms of computational time. Additionally, the 2-D SCNN loss function exhibits a consistent and monotonous decrease, with minimal instability, until it reaches the minimum loss value. This smooth and stable behavior is a key factor contributing to the rapid convergence of the SCNN model. In contrast, when considering the case of 2-D CNN, the loss function displays some instabilities during the early epochs. These instabilities can be attributed to the primary limitations of conventional CNN models in effectively handling non-Euclidean spaces, as evident in the denoising of noisy images.

For 3-D denoising example, the 3-D SCNN demonstrates comparable performance, and in the case of larger lesions, it exhibits superior quality and recoverability compared to the results obtained from iterative PET CNN. While the image contrast is marginally reduced in the 3-D SCNN results, it effectively preserves intricate edge details in the reconstructed images. Notably, the denoised SCNN sliced images exhibit reduced blurriness, particularly in the connecting regions of the contrasting lesions. We should note that the 3-D SCNN loss function follow similar pattern as the 2-D SCNN, however larger number of epochs was used to converge these results to compare with the 2-D SCNN. This is partially related to more intricate input dataset. Also 3-D SCNN is a newly developed package with a suboptimal simulation performance, and the limited number of representative functions currently available influences the computational performance at this stage.

Regarding assisted image reconstruction, SCNN-assisted approach shows high accuracy in reconstruction of rods with diameters ranging from 3.5 mm to 6.5 mm. However, the reconstruction is blurry for the rods with diameters of 2.2 mm and 1.6 mm. It is noticeable that the SCNN loss function consistently and steadily decreases with minimal instability until it reaches the minimum loss value, as depicted in Figure 5b. In contrast, the CNN-assisted results showcase accurate reconstruction of rods with diameters of 5.5 mm and 6.5 mm, while the 4.5 mm diameter rods are not well resolved, and the remaining rods are not well-resolved. The convergence rate of the CNN loss is significantly slower when compared to the SCNN loss. It is worth mentioning that increasing the number of epochs did not lead to improvements in CNN results. In terms of computational cost, the SCNN and CNN-assisted reconstructions take 870 seconds and 821 seconds, respectively, to complete 60,000 epochs. Notably, SCNN achieves convergence in just 1,000 epochs with a computational time of 15 seconds. Therefore, the computational cost is significantly reduced when compared to list mode MLEM, where each iteration takes approximately 2,112 seconds to compute on one CPU thread.

Overall SCNN offers a simple and straightforward architecture with few adjusting parameters that perform more efficient than the conventional CNN. We showed that it can be used as a trusted network for both 2-D and 3-D image processing problems and we also demonstrated SCNN can be used in the conjugation with the conventional image reconstruction techniques to improve the computational cost and output accuracy. This suggests that SCNN serves as a robust alternative to CNN in machine learning-based image processing.

## V. Conclusion

In this work, we demonstrated the effectiveness of equivariant SCNNs in improving image quality for various examples such as denoising and image reconstruction of 2-D benchmark brain image, while significantly reducing computational costs compared to conventional CNNs. We also extend our model to 3-D and examined the performance of newly developed 3-D SCNN on 3-D noisy dataset. Additionally, our study showcased the successful combination of equivariant SCNN with image reconstruction techniques such as list mode MLEM reconstruction, achieving high accuracy and computational efficiency even when utilizing a small subset of data in SCNN reconstruction. These findings highlight the potential of SCNN for broader medical imaging applications, especially in scenarios where input data may be limited and involve a spherical field of view (FOV) [28–30] and particularly in modalities with omnidirectional outputs like brain PET or cardiac dedicated scanners. Hence, further investigations are planned to explore the applicability of SCNNs for such problems in the future.

## Figures and Tables

**Fig. 1.**
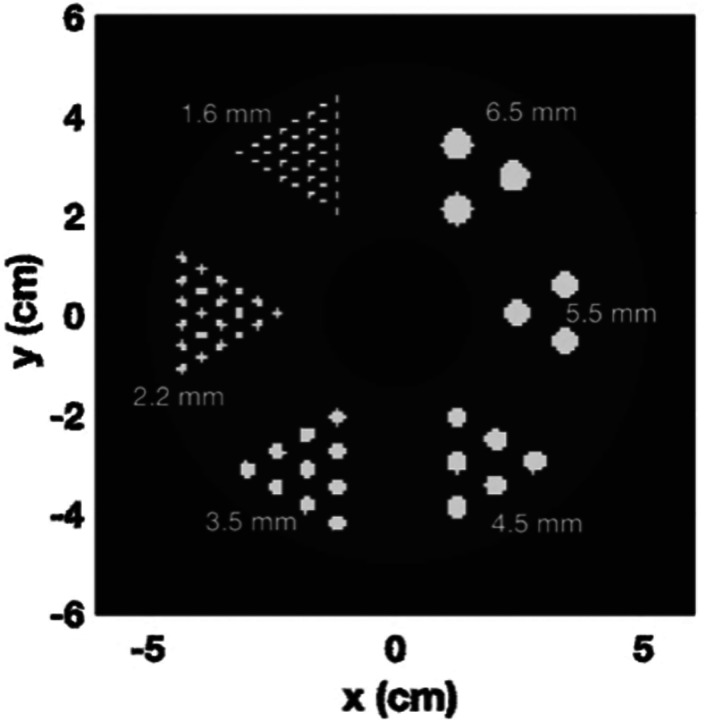
The designed phantom consists of 6 regions of hot rods.
